# Moving beyond the pros and cons of automating cognitive testing in pathological aging and dementia: the case for equal opportunity

**DOI:** 10.1186/s13195-014-0058-1

**Published:** 2014-08-29

**Authors:** Keith A Wesnes

**Affiliations:** 1 Wesnes Cognition, Little Paddock, Streatley Hill, Streatley on Thames, RG8 9RD, UK; 2 Department of Psychology, Northumbria University, Ellison Place 2, Newcastle-upon-Tyne, NE1 8ST, UK; 3 Centre for Human Psychopharmacology, Swinburne University, John St, Melbourne 3122, VIC, Australia

## Abstract

The lack of progress over the last decade in developing treatments for Alzheimer’s disease has called into question the quality of the cognitive assessments used while also shifting the emphasis from treatment to prophylaxis by studying the disorder at earlier stages, even prior to the development of cognitive symptoms. This has led various groups to seek cognitive tests which are more sensitive than those currently used and which can be meaningfully administered to individuals with mild or even no cognitive impairment. Although computerized tests have long been used in this field, they have made little inroads compared with non-automated tests. This review attempts to put in perspective the relative utilities of automated and non-automated tests of cognitive function in therapeutic trials of pathological aging and the dementias. Also by a review of the automation of cognitive tests over the last 150 years, it is hoped that the notion that such procedures are novel compared with pencil-and-paper testing will be dispelled. Furthermore, data will be presented to illustrate that older individuals and patients with dementia are neither stressed nor disadvantaged when tested with appropriately developed computerized methods. An important aspect of automated testing is that it can assess all aspects of task performance, including the speed of cognitive processes, and data are presented on the advantages this can confer in clinical trials. The ultimate objectives of the review are to encourage decision making in the field to move away from the automated/non-automated dichotomy and to develop criteria pertinent to each trial against which all available procedures are evaluated. If we are to make serious progress in this area, we must use the best tools available, and the evidence suggests that automated testing has earned the right to be judged against the same criteria as non-automated tests.

## Introduction

Cognitive dysfunction characterizes all of the dementias, and as a result cognitive testing is an essential element of all research in this field. Although numerous automated tests of cognitive function have long been available, the utilization of such procedures in clinical trials in the fields of pathological aging and dementia has not been widespread. Instead, the overwhelming majority of procedures currently employed are non-automated, many having been originally developed for use in clinical neuropsychology. This is exemplified by the Alzheimer’s Disease Assessment Scale-Cognitive subtest (ADAS-Cog), which has been the primary outcome in the approvals of the five currently approved drugs for Alzheimer’s disease (AD) [1] and in the trial upon which the US Food and Drug Administration (FDA) approved the first therapy for Parkinson’s disease dementia (PDD) [2].

During the last decade, the news from this field has been overwhelmingly negative; no new treatment has been approved for AD, despite a massive worldwide research effort which has been overshadowed by the failure of well over 100 putative treatments [3],[4]. This depressing experience has had two consequences relevant to this article. First, the ADAS-Cog has come under scrutiny for its effectiveness as an outcome measure in such trials, owing in part to failures to detect time-based declines in untreated patients [5], although other limitations have long been recognized (for example, [6],[7]). Second, in recent years, the focus of attention in therapeutic development in AD has turned to intervening at the prodromal or even preclinical stages of the disease [3],[8]-[10], areas in which the ADAS-Cog would have limited, if any, utility. As a consequence, a number of recent workgroups and consensus meetings had concluded that other more sensitive and appropriate assessments of cognitive function are required in the field [1],[9],[11],[12]. For instance, in 2010, the National Institute on Aging and the Alzheimer’s Association convened a workshop to discuss state-of-the-art methods for cognitive assessment, including computerized batteries, and new approaches under development [1]. The following quotes from the article set the scene for this review:

‘Computerized batteries offer a number of advantages over paper-and-pencil type tests, notably precise, accurate assessments that can be obtained with millisecond timing, ease of administration (sometimes with no administrator needed) and scoring, greater standardization, and adaptive presentation of items’.

‘Important disadvantages of computerized testing in older adults are that these tests can be challenging for people with visual limitations; they can be too fast-paced or difficult for people who are unfamiliar with computers; and participants may have problems adapting to a keyboard, mouse, or number pad’.

‘Yet more data are needed before computerized batteries can take the place of traditional assessments for clinical decision-making purposes. In addition, some people (both examiners and examinees) will just feel more comfortable with paper-and-pencil tests than computer-based batteries’.

The purpose of this review, as the title implies, is not to add fuel to the fire by arguing the relative merits of computerized testing over non-automated methods, but rather to attempt to level the playing field in order that tests, whether automated or not, will in the future be evaluated against a common set of criteria to determine their usefulness in clinical trials. As pointed out by Black and colleagues [12], ‘there must be a strategy to selecting a scale so that it measures what it is intended to measure. Whether the best measure turns out to be computer-based or pen-and-paper is then secondary’.

## Historical perspective

‘Pencil and paper’ tests, as in the quotation above and elsewhere (for example, [13]), are frequently termed ‘traditional’ in comparison with automated procedures. However, a pertinent and not widely recognized point is that automation of many of the cognitive tasks in use today preceded the development of the vast majority of tests used in clinical neuropsychology as well as the ADAS-Cog and Mini-Mental State Examination (MMSE) [14]. This mischaracterization of many computerized tests as ‘new kids on the block’ serves to undermine them, implying that they are ‘a work in progress’ or ‘experimental in nature’ or ‘have not yet stood the test of time’.

## Electromechanical solutions

In 1868, Franciscus Donders launched the field of ‘mental chronometry’ by developing the original versions of two now widely recognized tests: simple reaction time and choice reaction time (CRT) [15]. He devised and used the ‘noematachograph’, an instrument involving a helically moving horizontal cylinder mounted with ebonite and brass disks, electrodes, and a tuning fork. This ingenious apparatus could record the time lag between a stimulus (colored light, letter-symbol, or sound) and either manual or vocal responses. He demonstrated that CRTs were longer than simple reaction times, reflecting the extra information processing necessary to perform CRT, a finding which remains one of the central tenets of cognitive neuroscience. Since then, these tests, together with other assessments, including vigilance tasks, have been the mainstay of attention testing in cognitive neuroscience [16].

In 1919, the American Committee for the Study of the Tobacco Problem commissioned an investigation entitled ‘Tobacco and Mental Efficiency’. This program was led by the experimental psychologist Clark L Hull at the University of Wisconsin [17]. Hull, renowned for his experimental rigor and sophisticated approach to test validation [18], devised a series of cognitive tests to measure the effects of tobacco smoking on both smokers and non-smokers. One of the tests involved the speed with which visually presented four-letter words could be vocalized. Another involved the formation of connections between visually presented shapes and the vocalization of nonsense syllables, which today would be termed paired-associate learning. The sophistication of the instrumentation is illustrated by the following:

‘The reaction-time was measured by a Johns Hopkins chronoscope controlled by a tuning fork of special construction. The rate of this fork was calibrated against a Jaquet chronograph. It was such that the units of the chronoscope readings were 0.0034 of a second or approximately 1/300th. The chronoscope was connected electrically with the exposure apparatus in such a way that the instant a word came into view, the timing part of the chronoscope was automatically set going. When the subject spoke the word, a sensitive voice key automatically stopped it’.

Thus, over 90 years ago, a procedure had been developed to measure oral reaction times with a precision of 3.4 milliseconds, something not achieved in many currently available tests. This early clinical trial demonstrated that Hull’s methodology was sufficiently sensitive to detect cognition enhancement, and tobacco administration was found with a ‘fair probability’ to produce an improvement in speed on the reading reaction time and paired-associate learning tasks.

A range of other tests were developed in the middle of the last century. In 1948, Norman Mackworth developed a prolonged visual vigilance task to simulate monitoring a radar screen [19], which was shown to be sensitive to the effects of amphetamine [20], cigarette smoking, and nicotine tablets [21]. In 1956, Rosvold and colleagues [22] first described the continuous performance test (CPT), a paradigm in wide use today. It was used to study attention deficits in brain-damaged patients, and the motivation for developing the test was that current non-automated tests of attention, such as the Wechsler digit span and digit symbol substitution subtests, had ‘not consistently showed decline following brain damage’ [22]. The stimuli were letters presented on a rotating drum via a visor, a response key being mounted to the right. The authors concluded that brain-damaged patients had poorer performance on this task requiring continuous attention, and in 1959 the drug sensitivity of the CPT was confirmed [23]. Two other examples of electromechanical tests were developed in the early 1950s: (1) the Bakan test, a 48-minute sustained attention task in which a series of digits was presented via headphones at the rate of one per second, the participant having to detect sequences of three consecutive odd digits [24]; and (2) the pursuit rotor task, which involved using a stylus to follow a small disc of light on a turntable rotating at one revolution per second [25].

## Computerized cognitive tests

One of the pioneers in using the early computers to run cognitive tests was John Gedye, who demonstrated that such procedures could usefully assess the cognitive deficits in patients with brain damage and dementia [26]. All of the tasks described in the previous section have since been computerized over the last 50 to 60 years; the sensitivity of the Mackworth clock, CPT, and Bakan (now widely known as the rapid visual information processing task) have been enhanced by the additional collection of reaction times. The early laboratory computers were also used to develop new procedures such as the still widely employed memory scanning task [27]. The field really started to progress with the advent of the microcomputer in 1977 and the personal computer in the early 1980s. Since then, a huge variety of computerized tests and test systems have been developed and used widely in cognitive neuroscience and clinical trials (for example, [28],[29]), and many have been specifically designed to assess patients with mild cognitive impairment (MCI) and AD (for example, [1],[12],[30],[31]).

## Problems with computerized tests

Widely expressed potential downsides of computerized testing of older patients center on the capability of such individuals to operate computers and on the acceptability of such ‘novel’ procedures including the potential anxiety and stress individuals may experience when confronted with complex-looking equipment [1]; of course, it should be accepted that test anxiety has long been associated with non-automated tests [32],[33].

Sano [34] was one of the first researchers to recognize that attention deficits were prevalent in diseases of aging, including AD and Parkinson’s disease. A computerized two-choice reaction time task was developed for use by ‘mildly impaired’ patients with AD. Sano recognized the advantages of using a personal computer, including consistent presentation of task information, ease and accuracy of recording responses, and rapid data analysis. Given the era, it was acknowledged that the participants needed to become comfortable with the testing procedures; and the benefits were recognized of having the tasks explained and demonstrated by an administrator, as opposed to the participant having to follow on-screen instructions. Further requirements were having the on-screen stimuli presented at the size necessary to overcome any visual difficulties and for the responses to be simple and not require excessive strength. The benefits of practice on the task were acknowledged, and providing extra practice to prevent slower learning due to memory problems of the participants was required. The procedure was successfully implemented, and reaction time was found to be slower in older healthy participants than young participants and to be slowest of all in the AD group. The data permitted the conclusion that patients with AD have deficits to selective attention.

Frydenberg [35] reported on the utilization of the microcomputer in rehabilitation programs for older patients, which had begun in her institution in 1981. On the issue of whether there would be fear, resistance, or an inability to master the necessary skills, Frydenberg reported an overwhelmingly positive response by the patients. A similar response was seen in an early memory clinic evaluation of a computerized cognitive test system. The study established that dementia patients who were up to 94 years old and who had MMSE scores of as low as 6 could be tested satisfactorily [36]. The test system collected patient responses solely from a response box with two buttons, and previous development had determined optimal sizes and rates of presentation for the experimental stimuli. Two quotes from the article are revealing:

‘The utility of the testing in this population was very satisfactory, supported by the impression of the psychologist who administered the tests that patients of all abilities generally enjoyed performing the system … more than the other tests, many commenting that they hoped they would be able to perform it on another occasion’.

‘The clinic staff were surprised at the extent to which physically disabled patients including those with Parkinson’s disease and post-stroke patients were able to manage the response buttons’.

Another study, in a group of individuals who were at least 85 years old, sought to compare the relative acceptability of computerized tests administered via a tablet - simple reaction time (SRT), CRT, a vigilance task, and episodic verbal recognition - with ‘traditionally’ administered neuropsychological tests (the Wechsler digit symbol substitution word-list recall and word recognition tests) [37]. Testing was conducted in the homes of the participants, and it was found that only 91% could complete the neuropsychological tests but that 100% completed the computerized ones. The participants randomly assigned to computerized tests were significantly less likely to rate the tests as difficult, stressful, or unacceptable than those randomly assigned to pencil-and-paper tests. Researchers were also significantly less likely to rate participants as being distressed in the computer test group.

Although the above studies demonstrate that computerized testing can be acceptable to older patients and patients with dementia, are some methodologies more acceptable than others? One study contrasted two computerized cognitive test systems administered over the course of a 6-month period to AD patients who were up to 100 years old [38]. One, the Cognitive Drug Research (CDR) System, involved responses being made via a response box, and the other, the CANTAB (Cambridge Neuropsychological Test Automated Battery) System, involved the use of a touch screen. The study was unable to collect enough data on the latter test system because of difficulties, including unacceptability by the patients and problems with data storage, with the result that the research article on the study was based entirely on the findings of the CDR System. Another study, contrasting the cognitive deficit profiles of dementia with Lewy bodies (DLB) patients with AD patients, switched from the CANTAB System to the CDR System [39]. This was due to 90% of successive patient referrals being excluded from CANTAB testing because of problems with the procedural complexity, duration, nature, and the overall acceptability of the methods. In contrast, ‘the CDR battery used was specifically designed (Simpson *et al*., [36] 1991) for use with a demented population and this is reflected in the high (70%) inclusion rate of consecutively referred cases’ [39]. Various factors were cited as relevant to this higher utility, including the ability to temporarily pause tasks if patients became fatigued or distracted, the large size of the stimuli employed which minimized the impact of visual disturbances on task performance, the absence of any negative feedback about poor performance, and the brief nature of the tests. In contrast, only 30% of patients were excluded from being evaluated with the CDR System. Clearly, some methods of implementing automated cognitive testing to patients with dementia can prove unacceptable.

Other perceived limitations of computerized tests in the field of dementia may be the lack of regulatory acceptance of such procedures. In terms of regulatory precedent, in 1992, the FDA approved a computerized cognitive test system as the primary outcome variable in two pivotal phase III studies of the effects of D-Cycloserine in AD [40].

## Strengths of computerized tests

This section from a position paper published in 1997 by the International Working Group on Harmonization of Dementia Drug Guidelines [31] is pertinent:

‘Computerized procedures currently are used extensively in general psychopharmacology, and some systems have been developed specifically for use with demented patients. There is evidence that, after an initial familiarization, properly implemented computerized procedures can be perfectly acceptable to AD patients (Ferris *et al*., 1988; Simpson *et al*., 1991). Automated testing can have clear advantages for clinical trials in this field. The task information always is presented in a standard fashion; the recording of responses is done automatically and precisely, without any bias; and there are no grey areas involving differences in interpretation. These advantages can reduce variability both from session to session for a patient, and also between different national and international sites. Automated procedures recently have been shown to be more sensitive than the standard tests that are used extensively in this field (Mohr *et al*., 1996), and the sensitivity to anticholinesterases in patients with AD also has been established (Siegfried, 1993). Given the previously noted importance of assessing attention and processing speed in patients with AD, computerized tests can provide optimal procedures for assessing changes in these functions (for example, Wesnes *et al*., 1987; Nicholl *et al*., 1995). Some tests of attention such as vigilance can be run only on computers. However, before being used in major trials, extensive assessments of reliability, validity, and utility of these tests must be made. In addition to the aforementioned basic test criteria, crucial requirements for automated testing include the recording of responses via simple response buttons or touch screens, not the keyboard; absence of unwanted negative feedback from the tasks; timing routines that are accurate to the nearest millisecond and that are made independently of the internal clocks of the computer; established reliability of software and hardware; presentation of information using specially constructed fonts that are clearly visible to patients with AD, and security of automatically recorded data files that can be accessed only by authorized site staff members. The Work Group concluded that computerized procedures initially should be used together with the established procedures in the field (for example, the ADAS) so that the comparable utility and sensitivity of the two types of testing can be identified. If clear advantages of computerized procedures are demonstrated, such procedures might supersede existing methods’.

It should be noted that one of the authors of this article helped to develop the ADAS-Cog.

One of the more important advantages of automated testing is the comprehensive evaluation of task performance. A study found patients with MCI to have accuracy scores on working memory and episodic recognition tasks which were the same as those of non-impaired individuals; yet when the speed of the responses in the tasks was evaluated, deficits which approached or reached the levels of patients with dementia were found [41]. In regard to these findings, the authors commented:

‘It may be that patients in this group are aware of some deficit and know that, in a variety of situations, they have to work more slowly in order to achieve their main aim—the correct answer—a trade-off between accuracy and speed. Alternatively, they may have a deficit in the speed with which they can recognize and process information, although their accuracy of recognition is not much impaired. Thus, the speed of memory performance may be the first aspect of the memory system to decline as the system begins to fail. Other factors such as confidence and certainty may also be important’ [41].

Whatever the reason for this interesting finding, it would clearly have gone unnoticed with non-automated testing of working and recognition memory. Assessing reaction times on attention tasks has also been beneficial; for example, Ballard and colleagues [42] were able to discriminate DLB and PDD patients from AD patients on the basis of a selective prolongation of CRT in relation to SRT as well as an increase in the variability of reaction times. These differences reflected the bradyphrenia and fluctuating attention which are seen in DLB and PDD [43] and supported an earlier finding using computerized testing which identified bradyphrenia in Parkinson’s disease as being possibly related to dementia [44]. Mohr and colleagues [6] found computerized testing to be superior in correctly classifying patients to have either AD or Huntington’s disease in comparison with the ADAS-Cog, MMSE, Mattis Dementia Rating Scale, and the Wechsler Memory Scale. Furthermore, they found computerized testing to be best able to discriminate the two dementias. This more sophisticated profiling of cognitive dysfunction also confers benefits in the evaluation of drug therapy, one study enabling a fuller profile of benefits to be established than would be seen using neuropsychological tests [45], while another differentiated two of the registered anticholinesterases in AD patients on the basis of their relative effects on attention [46].

Can automation improve sensitivity in long-term clinical trials? One study evaluated the effects of an angiotensin II-receptor blocker on cognitive function over the course of 5 years in a population of 257 hypertensive, but not cognitively impaired, older adults with a mean age of 76 years [47]. It was found that, on computerized measures of episodic memory and attention, active treatment statistically significantly reduced the rate of decline seen under placebo over the 5-year period with effect sizes comparable to those seen with anticholinesterases in AD [48]. On the other hand, the neuropsychological tests used in this study did not identify any benefit (Trail Making test, Category Fluency, and Verbal Fluency). It should be noted that the overall study methodology and population in this study were comparable to those of trials in preclinical AD, suggesting that appropriate computerized testing will at the minimum be no less sensitive than neuropsychological tests.

Two recent examples illustrating the utility of automated testing in therapeutic clinical trials of pathological aging are relevant. Newhouse and colleagues [49] found automated testing, including the CPT, to help identify beneficial effects of nicotine over the course of 6 months in amnestic MCI, one of the few successful studies in this condition. A phase II study of a potent and selective alpha-2C adrenoceptor antagonist in 100 patients with moderate to moderately severe AD identified reliable benefits of the compound over the course of a 3-month period using a computerized test system [50]. On the basis of these results, a successful license agreement for the development and commercialization of the product has just been established with a partner company [51].

## Relative costs of using automated versus non-automated tests in clinical trials

It is worthwhile to consider the relative economics of the two types of testing. Certainly, there is a greater initial capital investment in providing study sites with computers. However, training the staff to administer automated and non-automated tests will require a comparable investment of time, and most computerized systems can be administered by non-specialist staff, whereas most neuropsychological tests need to be administered by suitably qualified personnel. Non-automated tests require manual scoring and data entry, which can be time-consuming and subject to human error, whereas the vast majority of computerized tests automatically record and score the data, and the study database is built up with relatively little staff involvement. Most tests, whether automated or not, are subject to license fees, and thus the overall costs to trials tend to balance out between the two methodologies, the higher initial outlay for providing computers being offset by the subsequent saving of time and personal involvement in data capture, scoring, and entry into the study database. Furthermore, the opportunities discussed later for remote data capture (for example, via the internet) can greatly reduce the costs and effort involved in test administration and data acquisition.

## Current status and future developments of the automation of cognitive tests

It should be clear from this review that automated tests have long been available for use in clinical trials in pathological aging and that a wide variety of systems are currently available. Various opportunities for gathering cognitive function data outside of the clinic or laboratory have also been explored. Since the start of the millennium, groups have conducted cognitive tests via a variety of remote platforms: via the telephone using interactive voice response technology [52], via cell phones (for example, [53]), and via the internet [54]. Furthermore, many neuropsychological tests have been computerized (for example, [55]), and digital pens enable any pencil-and-paper test to be automated (for example, [56]). Other developments have included virtual reality testing in AD patients [57] and cognitive testing embedded in games [58]. A computerized version of ADAS-Cog has also been developed and has been shown to have greater reliability than the original version [59]. However, to the knowledge of the author, this version has not been used in major clinical trials in the 3 years since that article appeared.

## Conclusions

A major thrust of this review has been that the fundamental requirements for computerized tests in terms of utility, reliability, validity, and sensitivity are no different from those for any other form of cognitive testing. Once these crucial properties are satisfactorily established for any test or test system, the overwhelming attribute which should drive test selection for therapeutic trials is the ability to reliably and sensitively measure change over time. The latter use of ‘reliably’ means that the test scores should reflect the cognitive abilities of the individual at each assessment time, not changes due to practice effects or noise in the assessment. Sensitivity, of course, means that any change, positive or negative, should be detectable with the highest possible levels of precision. There has been some historic prejudice toward highly sensitive measures, primarily in terms of the detection of changes which have little consequence to everyday life. However, over recent years, the reporting of effect sizes, such as in Cohen’s d, has become almost standard practice, thus enabling highly statistically reliable but trivial effects to be differentiated from effects of clinical and everyday relevance.

The present review has been an opportunity for the author to revisit a question posed 15 years ago:

‘The purpose of this chapter is to shed some light upon the intriguing question of *why*, for decades, some researchers have gone to such incredible lengths to harness the latest technological advancements of the day in order to automate tests of mental capabilities, while their colleagues have been perfectly happy to make such assessments simply using pencil, paper and sometimes also a stopwatch?’ [14].

It is hoped that the reader will now have a better understanding of the factors behind this interesting dichotomy and also that this review has convincingly illustrated that automated testing is by no means novel and that, with appropriate implementation, it can stand shoulder to shoulder with pencil-and-paper testing in terms of acceptability, utility, reliability, validity, and sensitivity. The intention has been to make the case in the strongest possible fashion that, for any particular trial, criteria should be established for the essential requirements of cognitive assessment to achieve the aims of the study and for these then to be applied evenly to all potential instruments available, whether they be computerized or pencil and paper.

## Abbreviations

AD: Alzheimer’s disease

ADAS-Cog: Alzheimer’s disease assessment scale-cognitive subtest

CANTAB: Cambridge neuropsychological test automated battery

CDR: Cognitive drug research

CPT: Continuous performance test

CRT: Choice reaction time

DLB: Dementia with Lewy bodies

FDA: US food and drug administration

MCI: Mild cognitive impairment

MMSE: Mini-mental state examination

PDD: Parkinson’s disease dementia

SRT: simple reaction time

## Competing interests

Until March 2014, the author was employed by Bracket (Wayne, PA, USA), which provides a computerized cognitive test system to the clinical trials industry.
